# Synbiotic enhances immune responses against infectious bronchitis, infectious bursal disease, Newcastle disease and avian influenza in broiler chickens

**Published:** 2015-09-15

**Authors:** Alireza Talebi, Amir Amani, Masoud Pourmahmod, Poya Saghaei, Reza Rezaie

**Affiliations:** 1 *Department of Poultry, Faculty of Veterinary Medicine, Urmia University, Urmia, Iran *; 2 *Graduate Student, Faculty of Veterinary Medicine, Urmia University, Urmia, Iran.*

**Keywords:** Avian influenza, Biomin Imbo, Infectious bronchitis, Infectious bursal disease, Newcastle disease

## Abstract

Increased susceptibility of birds to avian pathogens in intensive husbandry system has emphasized on necessity of improvement of innate and specific immune responses of birds by the fast establishment of a beneficial microflora and immune stimulator factors to guarantee healthy and low-price products. During this study, 192 one-day-old broiler chicks (Ross-380) in four groups with three replicates per group were used to investigate effectiveness of synbiotic Biomin Imbo on immune responses of the chickens following routine vaccination against Newcastle disease (ND), avian influenza (AI), infectious bronchitis (IB) and infectious bursal disease (IBD). The results of this study indicated that supplementation of Biomin Imbo in diet enhanced humoral immune responses significantly in the case of ND, IB, IBD (*p* = 0.049,* p* = 0.020, *p* = 0.036, respectively), but insignificantly in the case of AI (*p *= 0.160) following vaccination of the chickens against these most common important viral poultry diseases. It was more effective following vaccination with live than killed vaccines. In conclusion, application of synbiotic Biomin Imbo, as a feed-additive adjuvant promotes acquired humoral immune responses of broiler chickens.

## Introduction

In the 21^st^ century, immunization is still one of the most practical cost-effective prevention measures. Finding novel antigens as well as adjuvants is the most beneficial methods to induce an optimal protective immunity against human^1^ and poultry diseases including avian infectious bronchitis (IB), infectious bursal disease (IBD), Newcastle disease (ND) and avian influenza (AI) which cause significant economic losses in poultry industry worldwide.^2^^,^^3^ Interest in the dietary use of prebiotics and probiotics blossomed in the late 1800s/ early 1900 and the growing enthusiasm on the beneficial effects of pre-, pro- and synbiotics was motivated near the turn of the 20^th^ century.^4^ Ban of antibiotic growth promoters (AGPs) due to increased bacterial resistance and drug residues in poultry production together with consumer's demand for "natural" products have encouraged findings of alternatives for AGP. In order to preserve gut microbiota and to promote host innate defenses, administration of synbiotic (combinations of prebiotics, probiotics and immunomadulators elements) as alternative approach for promoting of performance and immune responses in modern poultry husbandry widely accepted.^5^^,^^6^ Probiotics affect the intestinal microbial balance and subsequently improve performance and reduce mortality in broiler chickens.^7^^,^^8^ Probiotics also protect chickens against avian pathogens,^9^^,^^10^ activate immunocytes and stimulate systemic immune responses^11^ including promoting the endogenous host defense mechanisms^12^ and enhancement of production natural antibodies^13^ as well as specific antibodies.^14^ On the other hand, prebiotics may control or manipulate microbial composition and/or activity, therefore combination of probiotics and prebiotics improve the survival rate of probiotics in digestive tract contributing to the stabilisation and/or enhancement of the probiotic effects.^15^^,^^16^ Under the present circumstances, improvement of post-vaccination immune responses against the most economically important poultry diseases, in particular IB, IBD, ND and AI is topic for research. Ideologically, synbiotics would have more beneficial effects than these elements alone.^17^^-^^20^ Therefore, the present project was undertaken to study the immuno-modulatory effects of the synbiotic Biomin Imbo on antibody responses during a routine vaccination of broiler chickens against IB, IBD, ND and AI as well as to compare its immunomodulatory effectiveness in vaccination with live and killed vaccines.

## Materials and Methods

**Chickens and experimental design.** One hundred and ninety-two one-day-old broiler (male and female) chicks (Ross-308 strain) were randomly allocated into four groups: (A) vaccinated + diet containing Biomin Imbo, (B) vaccinated + diet not-containing Biomin Imbo, (C) environmental control (unvaccinated + diet without Biomin Imbo), and (D) Biomin control (unvaccinated + diet containing Biomin Imbo). Three replicates were considered for each group (16 chicks per replicate). After leg labeling, the chicks of each replicate were housed in separated boxes and nutritional requirements (Ross-308, broiler nutrition), ambient temperature, lighting, ventilation as well as other environmental conditions fully met the requirements laid down in the technical instructions of Ross-308 broiler management.^21^ Vaccinated groups (A and B) and unvaccinated groups (C and D) were kept in separated houses.

Synbiotic Biomin Imbo containing of probiotic (*Enterococcus facium* IMB 52; 5 × 10^11^ CFU per kg), cell wall fragments of useful micro-organisms, prebiotic (fructo-oligosaccharides) and phycophytics (extracts of see algae) was used as recommended by manufacturer (Biomin GmbH, Herzogenburg, Austria). 

**Vaccine.** Vaccination was carried out according to the routine regional vaccination program. In the case of ND, based on optimal timing of maternal derived antibody (MDA) level (below log2^-3^), chickens of the groups A and B were vaccinated (live clone 30 vaccine, eye-drop) and (killed ND + AI vaccine, subcutaneously) on 11-days and second vaccination (only live vaccine) was carried out on 21-days of age using clone 30 strain of ND virus by eye-drop route as a recommended route inducing higher antibody titer with the closest-rang.^22^ In the case of AI, one vaccination is carried out on 11-days-old using killed (H9N2) vaccine by subcutaneous injection as a routine vaccination for broilers in the region. In the case of IBD, optimal time for first vaccination was estimated^3^ and D78 vaccine was used on day 16 and repeated on day 24 of age (based on MDA of the chicks). In the case of IB, as protection significantly (*p *< 0.05) correlated with levels of local respiratory antibody and not with serum antibody^23^ therefore, regardless to the potential negative effects of MDA against IB virus, Ma5 vaccine was used via eye-drop for vaccination of one-day-old chicks against IB on day 1 and is repeated on day 18 of age using the same vaccine.^2^^,^^24^

**Sampling.** As level of MDA titer is very important for determination of the best timing of vaccination against IBD as well as ND. On day 1 (one-day-old chicks), blood samples were collected from half the chicks of each replicate as previously described.^25^^,^^26^ On day 7 and then at weekly intervals (day 18 in the case of IB and day 24 in the case of IBD were exceptional) until 42 days of age, blood samples were collected from jugular veins and brachial vein, respectively as previously described.^27^^,^^28^ Blood samples were dated and labeled according to number of chickens. The collected sera were used to evaluate maternally-transferred antibodies of the chicks and to determine humoral immune response following vaccination against IB, ND, AI and IBD. 

**Serum antibody titers assessment.** Antibody level was determined using weekly serum samples of each bird separately in each replicate and treatment. Hemagglutination inhibition test (HI) was used for evaluation of antibody titers against ND and AI, as it has been reported that HI test is an excellent indicator of the immune status and disease resistance of a flock especially to assess protective response following vaccination,^27^^,^^28^ while the indirect enzyme-linked immunosorbent assay (IDEXX Laboratories Inc., Westbrook, Maine, USA) was used for evaluation of antibody titers as recommended for IB^29^ and IBD.^3^^,^^29^^,^^30^

**Statistical analysis.** SPSS software (Version 21; SPSS Inc., Chicago, USA) was used for analyzing of the results under completely randomized design employing one-way ANOVA analysis of variance and the means of different treatments were compared with Bonferroni, Duncan multiple range and repeated measure tests. Significance differences were taken at *p* < 0.05 level. 

## Results

**Fig. 1:**
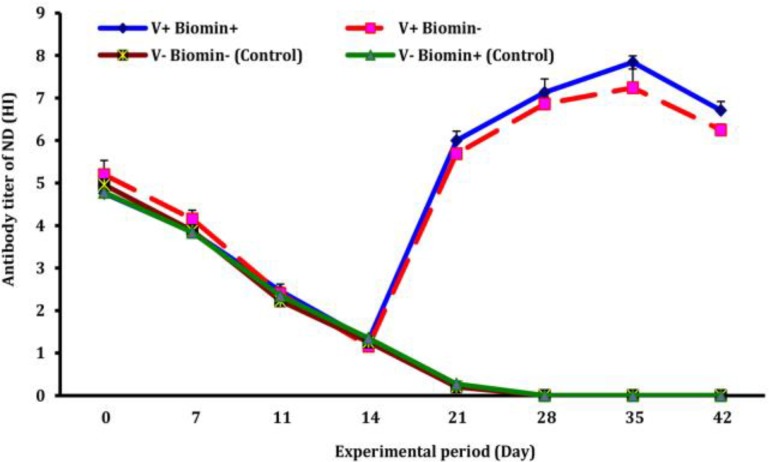
Effects of Biomin Imbo on Newcastle disease antibody titer of broiler chickens vaccinated with Clone 30 vaccine. V+ Biomin+ (vaccinated and fed with diet containing Biomin Imbo), V+ Biomin- (vaccinated and fed with diet without Biomin Imbo), V- Biomin- (unvaccinated and fed with diet without Biomin Imbo), V- Biomin+ (unvaccinated and fed with diet containing Biomin Imbo).

Newcastle disease antibody titer. Antibody titers against ND of the chickens of different groups are shown in Figure 1. As shown in this figure, maternally derived antibody (MDA) of the chickens gradually decreased in all the groups. Vaccination and feeding of Biomin Imbo did not affect the reduction rate of their MDA level. Antibody titers of vaccinated chickens started to increase at beginning of 3rd week (nearly 7 days post 1st vaccination), while those of unvaccinated chickens were steadily decreased. During this study, antibody titers of the vaccinated chickens peaked on day 35 of age, nearly two weeks post-2nd inoculation (pi), and the group treated with Biomin Imbo had the highest antibody titer and significantly (p = 0.049) differ when compared with those of only vaccinated chickens (Fig. 1).

**Fig. 2:**
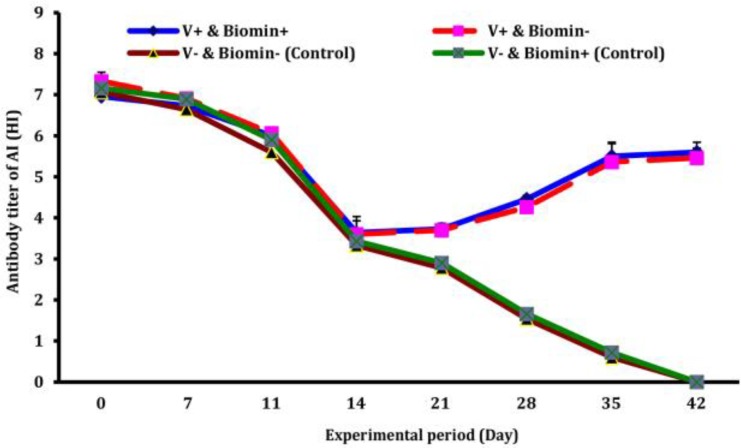
Effects of Biomin Imbo on avian influenza (AI) antibody titer of broiler chickens vaccinated with AI killed vaccine. V+ Biomin+ (vaccinated and fed with diet containing Biomin Imbo), V+ Biomin- (vaccinated and fed with diet without Biomin Imbo), V- Biomin- (unvaccinated and fed with diet without Biomin Imbo), V- Biomin+ (unvaccinated and fed with diet containing Biomin Imbo).

**Avian influenza antibody titer.** Antibody titers of the chickens against AI are shown in Figure 2. MDA of the control group gradually reduced and reach undetectable level around day 42, while those of vaccinated chickens increased steadily following vaccination and reached the highest level at six weeks age (around four weeks pi). Antibody titers of vaccinated chickens treated with Biomin Imbo had higher level in comparison to those of only vaccinated group (group B), although by aging (day 35 to day 42) differences between Biomin Imbo treated group (group A) and only vaccinated group (group B) is increasing (Fig. 2), but the difference was not significant (*p* = 0.160).

**Fig. 3:**
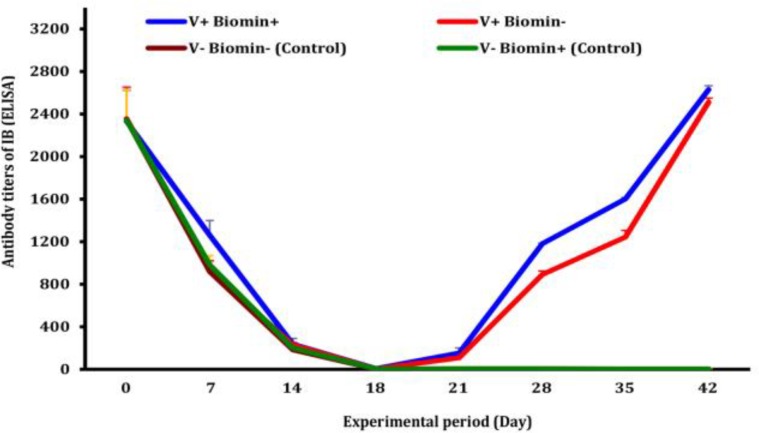
Effects of Biomin Imbo on infectious bronchitis antibody titer of broiler chickens vaccinated with Ma5 vaccine. V+ Biomin+ (vaccinated and fed with diet containing Biomin Imbo), V+ Biomin- (vaccinated and fed with diet without Biomin Imbo), V- Biomin- (unvaccinated and fed with diet without Biomin Imbo), V- Biomin + (unvaccinated and fed with diet containing Biomin Imbo

**Infectious bronchitis antibody titer.** Status of MDA and acquired antibody titer against avian infectious bronchitis are shown in Figure 3. MDA level of the chickens in all treatments gradually declined until 18 day of age and reduction of MDA of unvaccinated chickens continued to un-detectable level up to end of experiment.

On the other hand, antibody titers of vaccinated chickens increased gradually but steadily following 10 days post-2^nd ^vaccination and peaked around 42 day of age. In comparison to groups A and B (vaccinated groups), antibody titers of chickens treated with Biomin Imbo (group A) differed significantly (*p* = 0.020) from those of group B during day 28 to end of experiment (Fig. 3). 

**Fig. 4:**
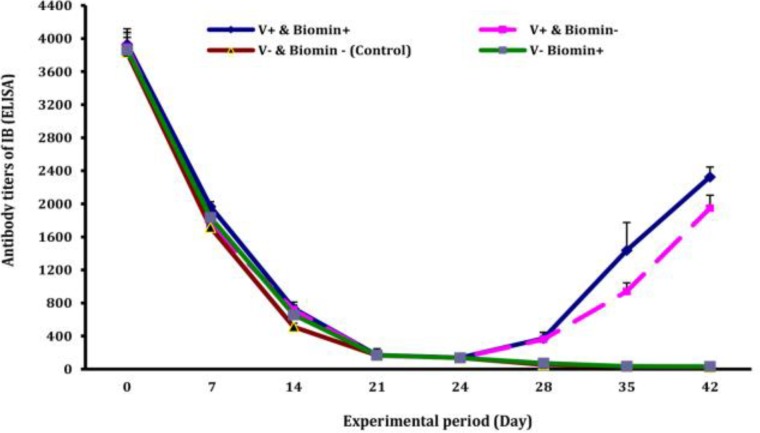
Effects of Biomin Imbo on infectious bursal disease antibody titer of broiler chickens vaccinated with D78 vaccine. V+ Biomin + (vaccinated and fed with diet containing Biomin Imbo), V+ Biomin- (vaccinated and fed with diet without Biomin Imbo), V- Biomin- (unvaccinated and fed with diet without Biomin Imbo), V- Biomin+ (unvaccinated and fed with diet containing Biomin Imbo

**Infectious bursal disease antibody titer.** Maternally derived antibody of the chicks together with acquired humoral immune responses following vaccination against IBD was shown in Figure 2. IBD disease's MDA of the chickens in all the groups declined according to half-life time (3 to 3.5 days) based on weight gain of broiler chickens and those of the unvaccinated groups (C and D) continued to wane until end of the experiment, indicating that neither environmental nor cross contamination occurred. However, antibody titer of vaccinated chickens increased following 2^nd^ vaccination. As shown in Figure 4, the chickens vaccinated and treated with Biomin Imbo diet had higher (*p* = 0.030) antibody titer than those of vaccinated but not treated with Biomin Imbo diet. 

## Discussion

In general, dietary supplementation of synbiotic Biomin Imbo not only ameliorate performance of poultry^31^^,^^32^ but also leads to immuno-modulation of humoral immune responses as well as cellular immune responses,^33^ however debates on their potential side effects (cytotoxic and moderate genotoxic effects) is open.^34^ Comparison of a growth promoters, prebiotics, probiotics as well as synbiotics on their preventive effects in colonization of *salmonella* in poultry revealed that antimicrobial agents allowed higher colonization as compared to prebiotics and probiotics,^9^ but Biomin controls the intestinal colonization of *Salmonella enteritidis* in chickens.^35^

With regards to immunomodulatory effects of Biomin Imbo, there are some reports that probiotic (*Enterococcus faecium*) of Biomin Imbo enhances humoral immune responses against sheep red blood cells (SRBC).^36^ Biomin Imbo also increases most parameters of blood profile including total protein^37^ and higher protein promotes induction of specific antibody titer against avian pathogens. Dietary inclusion of synbiotic Biomin Imbo increased growth performance and improved intestinal morphology, nutrient absorption^38^ and resistance of birds to pathogens or diseases.^37^ Comparison of the synbiotic with another probiotic indicated that the synbiotic had much more beneficial effects than probiotic alone^5^ as well as prebiotics alone^39^ or AGP alone.^40^ Enhanced effects of Biomin Imbo on antibody titers of the chickens against ND, AI, IB and IBD were observed during this study and is in agreement with previous reports that the serum antibody responses to oral and systemic administration of antigens were significantly enhanced by probiotics supplementation.^41^ Due to the immunomuadulatory effects of vitamin E, future synbiotics may include vitamin E as well.^42^

Continuously reduction of ND antibody titer of chickens of unvaccinated groups (C and D) and its remaining at undetectable level during experimental period confirmed that neither environmental nor cross contamination had occurred. Antibody titers of vaccinated chickens (groups A and B) increased following first vaccination (live + killed) and reached the highest level on day 35 of age (two weeks post 2^nd^ vaccination). Analyzing of the results, as shown in Figure 1, revealed that a) Differences among the groups (A, B, C, D) were not significant (*p* = 0.100) until 21 day of age; b) From day 21 up to end of the experiment, difference between vaccinated (A, B) and unvaccinated (C, D) groups due to vaccination was significant (*p* = 0.010); c) Comparison between antibody titers of chickens of group A (vaccinated and treated with Biomin Imbo) and those of chickens of group B (vaccinated but not treated with Biomin Imbo) was significant on the day 35 (*p* = 0.049) and on the day 42 (*p* = 0.048) of age. 

The results obtained during this study (Fig. 1) is in agreement with results as previously reported^37^^,^^43^ and could be attributed to the enhancement effects of Biomin Imbo on immune-inducing-cells. Average antibody titers of chickens group A (log2^-7.84^) is the highest available titer that could be induced by vaccinations (two live + one killed vaccines) as mean titers 4 to 6 log2 for single live and at least log2^-8^ for live plus killed vaccine was reported by OIE.^43^ Higher ND titers of chickens treated by Biomin Imbo is observed in our study is also in agreement with those of log2^-7.2 ^and mean titer of log2^-7.5 ^was reported for mentofin treated chickens.^44^ The beneficial effects of Biomin Imbo could be more evident in undesirable circumstances due to intensive husbandry systems. However, the enhancement effects of Biomin Imbo on humoral immune responses against ND observed during this study is also been reported for an another probiotics^14^ as well as other synbiotics.

As shown in Figure 2, influenza MDA of all groups waned gradually and those of unvaccinated chickens (group C and D) reached to undetectable level around 6 weeks of age with a half-life of 5.5 days as reported for broilers. Analyzing of the results obtained during this study revealed that: a) Differences among the groups (A, B, C, and D) were not significant (*p* = 0.150) until 21 day of age; b) From day 21 up to end of the experiment, difference between vaccinated (A, B) and unvaccinated (C, D) groups due to vaccination was significant (*p* = 0.020); c) Difference between group A (vaccinated and treated with Biomin Imbo) and group B (vaccinated but not treated with Biomin Imbo) was not significant (*p* = 0.160). Regarding lack of significant increasing effects of Biomin in the case of AI, it may be attributed to the mechanism of this product on providing a better condition for multiplication of live vaccines whereas a killed vaccine was used in the case of AI. High antibody titers observed during this study (Fig. 2) for chickens of groups A and B are good enough for one vaccination at six weeks of age following vaccination with inactivated H9N2 vaccine. Higher antibody titers (Mean titer of 2^-5.6^) of chicken group A could be attributed to enhancement effects of Biomin Imbo on antibody titers of the chickens as it has been reported that optimal nutritional status may enhance immune function indicated by increased vaccine response following vaccination against influenza.^37^^,^^45^

Humoral immunity has a key role in protection of chickens against IB.^24^ As shown in Figure 3, lack of serum antibody titer (nearly negligible until day 24 of age) could be explained that the MDA can interfere with the immune responses, but maternal antibody-positive chickens have a weaker virus-neutralizing antibody response to a second IBV vaccination compared to maternal antibody-negative chickens (*p* < 0.05).^33^ As maternal IBV antibodies are in low concentrations in the tear secretions than in sera, therefore, the interference between MDA and virus of vaccine may happen in a very low level. However, in the eye-drop or spray routes, invasion of the gland by virus of vaccine without the involvement of blood borne circulation after infection by the conjunctival and intranasal routes, would explain why the high levels of MDA of one-day-old chicks did not impair immunization.^46^ Lack of rising of antibody titers of unvaccinated chickens (groups C and D) during experimental period indicated that there was neither environmental nor cross contamination. Late rising of antibody titer (28 days post-1^st^ vaccination and 10 days post-2^nd^ vaccination) of the vaccinated chickens (groups A and B) and reaching the highest level at six weeks age were observed during this study is in agreement with the studies reporting that antibody peaked around 45 day of age following vaccination on day 1 and on day 25. Our observation on enhancement effects of Biomin Imbo on humoral response against IB is in agreement with the results reported for an another synbiotic.^33^ Recent studies indicate that supplementation of vitamin E may also enhances higher immune responses against IB.^42^

Humoral antibody plays a key role in protection against IBD.^3^ Maternally-derived antibody transferring rate (up to 73.00%) from breeders to yolk/chicks not only varied among different chickens' line but also MDA varied among one-day-old chicks even from same broiler breeder flock^47^ and depending on the range of MDA, finding optimal timing of primary vaccination would be too difficult. Although there are several methods for predicting the timing of initial vaccination,^30^^,^^48^ in routine vaccination program for intensive poultry husbandry system, primary vaccination against IBD may equalized MDA of the chicks and good immune response may be obtained following booster dose. Generally, infectious bursal disease with clinical signs occurs around three to six weeks of age^3^ and the birds are most susceptible at 30 to 35 days old. Therefore, an ideal vaccination program must induce protective antibody titer at this age as occurred during this study (Fig. 4) and IBD titer obtained from vaccination is able to protect the birds on susceptible ages as it has been reported that antibody titers over 1500 protected birds from very virulent IBD virus.^49^ As shown in Figure 4, MDA of the control group had completely declined by 42 days of age and this observation is in agreement with previous reports depending on the MDA level of birds.^47^ Additionally, the vaccination did affect the reduction rate of MDA as shown in Figure 4. There is always a critical period of gap between decay of passive immunity (i.e., MDA) and active immunity induced by vaccination but duration of the gap is depending upon type (degree of attenuation) of IBD vaccines (gap is longer in intermediate than intermediate plus vaccines) and MDA level (gap is longer in higher MDA than in lower MDA).^50^ The immunity gap problem could be solved by using of immune complex vaccines or DNA vaccines or vectored vaccines or intermediate plus IBD vaccines.^50^ Analyzing of the results showed that: a) Differences among the groups (A, B, C, D) were not significant (*p* = 0.100) until day 28 of age; b) From day 28 up to end of the experiment, difference between vaccinated (A, B) and unvaccinated (C, D) groups due to vaccination was significant (*p* = 0.020); c) Difference between group A (vaccinated and treated with Biomin Imbo) and group B (vaccinated but not treated with Biomin Imbo) was significant (*p* = 0.036) only on day 42 of age. 

In conclusion, Application of synbiotic Biomin Imbo enhances antibody responses following vaccination against ND, AI, IB and IBD but it is more effective in live than killed vaccines and could be used as a feed-additive adjuvant for improving innate and acquired immune responses in chickens.
